# TP53 Mutation as Potential Negative Predictor for Response of Anti-CTLA-4 Therapy in Metastatic Melanoma

**DOI:** 10.1016/j.ebiom.2018.05.019

**Published:** 2018-05-22

**Authors:** Wenjing Xiao, Nan Du, Taoyuan Huang, Jinan Guo, Xingkui Mo, Tao Yuan, Yong Chen, Ting Ye, Chunwei Xu, Wenxian Wang, Guoqiang Wang, Shangli Cai, Jing Chen

**Affiliations:** a Department of Tumor Radiotherapy, The Affiliated Hospital of Qingdao University, No. 16, Jiangsu Road, Shinan District, Qingdao, PR China; b Department of Oncology, The First Affiliated Hospital, Chinese PLA General Hospital, No. 51, Fucheng Road, Beijing, PR China; c Department of Dermatologic Surgery, Dermatology Hospital of Southern Medical University, Guangdong Province Dermatology Hospital, No. 2, Lujing Road, Yuexiu District, Guangdong Province, PR China; d The Department of Urology, The Second Clinical Medical College of Jinan University (Shenzhen people's Hospital), Shenzhen Urology Minimally Invasive Engineering Center, Shenzhen, PR China; e Department of Orthopaedics, The Third Affiliated Hospital of Kunming Medical University, Tumor Hospital of Yunnan Province, No 519, Kunzhou Road, Xishan District, Kunming, Yunnan Province, PR China; f Department of musculoskeletal surgery, Fudan University Shanghai Cancer Center, Department of Oncology, Shanghai Medical College, Fudan University, Shanghai, PR China.; g Cancer Center, Union Hospital, Tongji Medical College, Huazhong University of Science and Technology, Wuhan, PR China; h Department of Pathology, Fujian Cancer Hospital, Fujian Medical University Cancer Hospital, Fuzhou, Fujian, PR China; i Department of Chemotherapy, Zhejiang Cancer Hospital, Hangzhou, Zhejiang, PR China; j The Medical Department, 3D Medicines Inc., Shanghai, PR China

**Keywords:** Anti-CTLA-4, TP53, Melanoma, Biomarker, Tumor mutational burden

## Abstract

TP53 has been proved to be associated with cytotoxic T-cell induced apoptosis, however, the association between TP53 and the benefit of immunotherapy in melanoma has not been studied. In the present study, we examined the relationship between TP53 mutation and response to CTLA-4 blockade in metastatic melanoma by analyzing the data from one public cohort consisting of 110 patients with metastatic melanoma. The sequencing, mRNA and survival data of 368 patients with skin melanoma from The Cancer Genome Atlas (TCGA) was used to explore the underlying mechanism. TP53 mutation was associated with significant poorer progression-free survival (HR, 2.25; 95% CI, 1.15–4.37; P = 0.014), poorer overall survival (HR, 2.05; 95% CI, 1.02–4.13; P = 0.040) and trend of poorer response (OR, 0.20; 95% CI, 0.02–1.62; P = 0.131). The correlations were significant in multivariate analysis including lactate dehydrogenase, tumor mutational burden and tumor stage (P < 0.05). In TCGA, no association was observed between TP53 mutation and survival (P = 0.55). The mRNA expression of FAS was lower in patients with TP53 mutation than TP53 wild-type. Our findings suggest that TP53 mutation is a potential negative predictor of metastatic melanoma treated with CTLA-4 blockade.

## Highlights

• TP53 mutation is associated with poorer outcomes in patients with metastatic melanoma receiving anti-CTLA-4 treatment.
• The mRNA expression of FAS was lower in patients with TP53 mutation than TP53 wild-type.
• TP53 is a potential negative predictor of metastatic melanoma treated with CTLA-4 blockade.

Immune checkpoint blockades by enhancing the anti-tumor activity of immune system have been standard treatment for several advanced tumors, however, only a subset of patients can benefit from them. In this study, we find patients with melanoma harboring TP53 mutation show poorer survival outcome and response of anti-CTLA-4 treatment. The mRNA expression of FAS is lower in patients with TP53 mutation, suggesting that TP53 down-regulates the expression of FAS and impede cytotoxic T-cell induced apoptosis in melanoma. These results suggest that TP53 mutation may serve as a negative predictor of anti-CTLA-4 treatment in melanoma.

## 1 Introduction

In the last decade, immune checkpoint blockades (ICBs) of targeting cytotoxic T-lymphocyte antigen-4 (CTLA-4), programmed death receptor-1 (PD-1) and its associated ligand (PD-L1) as monotherapy or combination therapy, have revolutionized the treatment of metastatic melanoma [[1], [2], [3], [4]]. Unfortunately, only a subset of treated patients responds to the current immunotherapy. Therefore, it has become a challenge to identify clinically useful biomarkers that can distinguish patients who may respond or resist to ICBs.

Biomarkers including PD-L1 expression, tumor mutational burden (TMB), tumor-infiltrating lymphocytes, micro-satellite instability and immune gene signatures have been shown to be associated with the clinical benefit of ICBs [5]. Among these biomarkers, TMB is associated with better clinical outcome in metastatic melanoma treated with anti-CTLA-4 or anti-PD-1 therapy [[6], [7], [8]]. However, high TMB cannot guarantee response to ICBs, suggesting that other independent variables may exist to predict the clinical outcome beyond the existing biomarkers.

TP53, a well-known tumor suppressor and transcriptional activator or repressor, is the most frequently mutated genes in cancer [9], mutation of which allows tumor evasion and induces rapid tumor progression [10]. Preclinical studies have illustrated TP53-induced enhancement of cytotoxic T-cell (CTL) response by up-regulating major histocompatibility complex I (MHC I) and FAS [11,12]. In MHC antigen presentation pathway, oligopeptides generated via proteasome degradation require transporter associated with antigen processing 1 (TAP1)-mediated transport to rough endoplasmic reticulum and endoplasmic reticulum aminopeptidase 1 (ERAP1)-mediated trimming to fit the length criterion for MHC I presentation. The transcription of both TAP1 and ERAP1 are directly modulated by TP53, and the inactivity of mutant TP53 decreased their de novo protein synthesis and thereby the surface level of MHC-peptide complex, resulting in down-regulated immune surveillance [13,14]. Moreover, the gene of FAS is also targeted by the transcriptional activation of TP53. Thus, mutant TP53 diminishes its surface level in tumor cells and therefore inhibits CTL-induced apoptosis [12]. However, in a recent clinical study of lung adenocarcinoma, TP53 has been shown to be associated with higher TMB and better outcome of anti-PD-1 therapy [15]. The predictive value of TP53 mutation in patients treated with ICBs seems to be controversial and needs to be further illustrated.

In order to demonstrate the association between TP53 mutation and clinical outcome of ICBs, we analyzed the data from the largest available cohort of metastatic melanoma treated with anti-CTLA-4 with both genomic and clinical data [16]. The sequencing, mRNA and survival data from The Cancer Genome Atlas was also analyzed to explore the possible underlying mechanism.

## 2 Materials and Methods

### 2.1 Clinical Cohorts

The whole-exome sequencing and clinical data of 110 patients with metastatic melanoma treated with ipilimumab were obtained from the public cohort [16]. The Allen cohort is the largest cohort available in melanoma treated with ICBs with both genomic and clinical data. The genomic, survival and mRNA data of 368 patients with skin cutaneous melanoma (SKCM) was obtained from The Cancer Genome Atlas (TCGA) (www.cbioportal.org). The estimated CD8^+^ T cell infiltration data of TCGA samples was obtained from a previous study [17]. Most of the patients enrolled in TCGA were early stages.

### 2.2 Study Design

Any mutation in coding region of TP53 was determined as TP53 mutation. TMB was defined as the total number of nonsynonymous mutation in coding region. TMB-high group was defined as TMB ≥ median, while TMB-low group was defined as TMB < median. Indel burden was defined as the total number of insert and deletion mutation in coding region. The primary outcome was PFS, which was calculated from the date of first immunotherapy administration to disease progression or death due to any cause. The secondary outcome was OS, which was calculated from the date of first immunotherapy administration until death due to any cause and response rate. We first determined the association between TP53 and PFS or OS using univariate and multivariate models. Then we examined the association between TP53 and response in univariate and multivariate models. As previously reported, patients were stratified into response groups based on RECIST criteria [18], duration of OS and duration of PFS [16].

### 2.3 Statistical Analyses

Data analyses were performed using SPSS 20.0 (SPSS Inc., Chicago, IL). Survival description was illustrated by the Kaplan-Meier curves, with P value determined by a log-rank test. Hazard's ratio (HR) was determined through the univariate and multivariate Cox regression. The associations between response and variables were examined by a univariate logistic regression. Variables with significant P values or interest were included into multivariate logistic regression. Continuous variables were compared by Mann-Whitney *U* test. False discovery rate (FDR) was used to estimate the significance of differences between the mRNA expression levels. All reported P values were two-tailed, and P < 0.05 and FDR < 0.05 is considered statistically significant.

## 3 Results

### 3.1 Patient Cohort

110 patients with metastatic melanoma treated with anti-CTLA-4 were included in this analysis. The baseline characteristics were summarized in Table 1. 78 of 110 patients (70.9%) were male and the median age of the cohort was 61.5 years (range, 18 to 86 years). Most patients (90.9%) were stage 4. The median TMB count was 203.5 (range, 12–5976). Patients with TMB ≥ median were defined as TMB-high. 34 patients (30.9%) harbored BRAF^V600E^ mutation and 10 patients (9.1%) carried TP53 mutation including 6 missense mutation, 3 nonsense mutation and 1 splice site mutation.

**Table 1:** Baseline characteristics.

Variable	Total (n = 110)
Sex, n (%)	
Male	78 (70.9%)
Female	32 (29.1%)
Age, median (range), years	61.5 (18–86)
Stage, n (%)	
Stage 3	10 (9.1%)
Stage 4	100 (90.9%)
Primary, n (%)	
Skin	92 (83.6%)
Occult	14 (12.7%)
Mucosal	4 (3.6%)
Baseline LDH, n (%)	
Normal	58 (52.7%)
Abnormal	48 (43.6%)
Unknown	4 (3.6%)
Tumor mutational burden, median (range)	203.5 (12-5976)
BRAF^V600E^ mutation	34 (30.9%)
TP53 mutation	10 (9.1%)

LDH, lactate dehydrogenase.

### 3.2 Association Between TP53 and Survival Outcomes of Anti-CTLA-4 Therapy

**Fig. 1:**
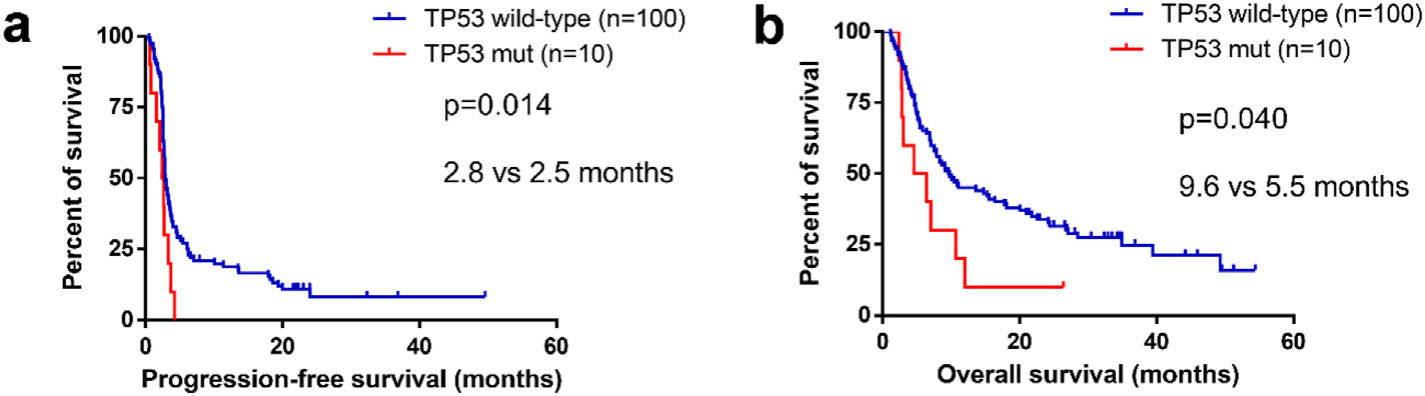
Association between TP53 mutation status and survival outcomes. (a) Kaplan-Meier survival curves of progression-free survival comparing patients with TP53 wild-type and patients with TP53 mutation. (b) Kaplan-Meier survival curves of overall survival comparing patients with TP53 wild-type and patients.

The detailed baseline characteristics and clinical outcomes of patients with TP53 mutation were summarized in Supplemental Table S1. We first analyzed whether TP53 mutation status was associated with the survival outcomes of anti-CTLA-4 in metastatic melanoma. Patients with TP53 mutation obtained poorer PFS compared to wild-type TP53 (median PFS: 2.5 months vs. 2.8 months, P = 0.014, Fig. 1a). Previous studies have demonstrated that TMB and LDH are associated with the clinical benefit of ICBs in melanoma ([7,16,19]. In the present study, univariable analysis revealed significant association between poorer PFS and LDH-abnormal or TP53 mutation, while no relation between PFS and TMB as continuous variable or binary variable with median as cut-off (Table 2). In a multivariate model including tumor stage, LDH, TMB and TP53, TP53 mutation and LDH-abnormal remained an independent prognostic indicator for poorer PFS (Table 2). TMB ≥ median showed borderline improvement in PFS (HR, 0.69; 95% CI, 0.45 to 1.04; P = 0.077; Table 2).

**Table 2:** Univariate and multivariate analyses of progression-free survival.

Parameter	Univariate analysis			Multivariate analysis		
	HR	95% CI	P	HR	95% CI	P
Age	1.00	0.99 to 1.01	0.935			
Sex						
Male vs. female	0.93	0.60 to 1.44	0.740			
Stage						
Stage 4 vs. stage 3	2.38	1.10 to 5.17	0.028	2.04	0.93 to 4.45	0.074
Tumor mutational burden						
Continous variable	1.00	1.00 to 1.00	0.604			
≥ median vs. < median	0.86	0.58 to 1.28	0.457	0.69	0.45 to 1.04	0.077
Indel Burden						
≥75th percentile vs. <75th percentile	1.15	0.75 to 1.76	0.530			
LDH						
Abnormal vs. normal	2.03	1.35 to 3.05	0.001	2.48	1.61 to 3.81	<0.001
TP53						
Mutation vs. wild-type	2.25	1.15 to 4.37	0.017	3.49	1.68 to 7.24	0.001
BRAF^V600E^						
Mutation vs. wild-type	0.87	0.56 to 1.35	0.534			

LDH, lactate dehydrogenase.

In consistence with PFS, poorer OS was discovered in patients with TP53 mutation compared to patients with wild-type TP53 (median OS: 5.5 months vs. 9.6 months, P = 0.04, Fig. 1b). Univariate and multivariate analysis exposed significant association between OS and TP53 status, LDH or stage (Table 3). No association was discovered between OS and TMB as continuous variable, while stratifying patients as per median TMB revealed signification relation between TMB ≥ median and better OS (Table 3), suggesting that TMB alone may not be sufficient enough to predict the clinical outcomes of anti-CTLA-4 treatment. In consideration of a previous study demonstrating that TP53 mutation is involved in the carcinogenesis in cutaneous melanoma [20], we further detected the correlation between TP53 mutation and clinical outcomes in the patients with skin melanoma (n = 92). The associations between TP53 mutation and survival outcomes were more pronounced (Supplementary Fig. S1).

**Table 3:** Univariate and multivariate analyses of overall survival.

Parameter	Univariate analysis			Multivariate analysis		
	HR	95% CI	P	HR	95% CI	P
Age	1.00	0.99 to 1.02	0.548			
Sex						
Male vs. female	0.78	0.49 to 1.24	0.294			
Stage						
Stage 4 vs. stage 3	0.22	0.07 to 0.69	0.010	4.04	1.27 to 12.89	0.018
Tumor mutational burden						
Continuous variable	1.00	1.00 to 1.00	0.206			
≥ median vs. <median	0.72	0.47 to 1.11	0.143	0.56	0.36 to 0.88	0.012
Indel burden						
≥75th percentile vs.	0.92	0.57 to 1.49	0.739			
<75th percentile						
LDH						
Abnormal vs. normal	2.07	1.33 to 3.22	0.001	2.58	1.61 to 4.14	<0.001
TP53						
Mutation vs. wild-type	2.05	1.02 to 4.13	0.044	3.27	1.52 to 7.02	0.002
BRAF^V600E^						
Mutation vs. wild-type	0.70	0.43 to 1.16	0.165			

LDH, lactate dehydrogenase.

### 3.3 Association Between TP53 Status and Response to CTLA-4 Blockade

The objective response or long-term benefit rate was 35% or 10% in patients with wild-type TP53 or patients with TP53 mutation patients respectively. In univariate logistic regression, patients with TP53 mutation exhibited a trend of poorer objective response or long-term benefit (OR, 0.20; 95% CI, 0.02 to 1.62; P = 0.131), whereas stage, LDH and TMB were associated with objective response or long-term benefit with odd's ratios of 0.10 (0.02 to 0.51), 2.50 (1.10 to 5.67) and 0.35 (0.15 to 0.83), respectively (Table 4). However, in multivariate logistic regression including TP53 status, stage, LDH and TMB, TP53 mutation was significantly associated with poorer objective response or long-term benefit with an OR of 0.11 (P = 0.049, Table 4). The inconsistence between univariate and multivariate logistic regression was likely due to the sample size of TP53 mutation group and potential confounding effects of TMB, LDH and stage.

**Table 4:** Univariate and multivariate analyses of response.

Parameter	Univariate analysis			Multivariate analysis		
	OR	95% CI	P	OR	95% CI	P
Age	1.01	0.98 to 1.03	0.720			
Sex						
Male vs. female	1.43	0.58 to 3.52	0.430			
Stage						
Stage 4 vs. stage 3	0.10	0.02 to 0.51	0.005	0.1	0.02 to 0.54	0.008
Tumor mutational burden						
Continuous variable	1.00	1.00 to 1.00	0.145			
≥ median vs. < median	2.50	1.10 to 5.67	0.028	4.61	1.52 to 10.89	0.005
Indel burden						
≥75th percentile vs.	1.86	0.79 to 4.36	0.153			
<75th percentile						
LDH						
Abnormal vs. normal	0.35	0.15 to 0.83	0.017	0.23	0.08 to 0.63	0.004
TP53						
Mutation vs. wild-type	0.20	0.02 to 1.62	0.131	0.11	0.01 to 0.99	0.049
BRAF^V600E^						
Mutation vs. wild-type	1.34	0.58 to 3.12	0.495			

### 3.4 Association Between TP53 and Survival Outcomes From TCGA

To further explore whether TP53 mutation is a predictive or prognostic biomarker for melanoma, we retrieved the survival data from The Cancer Genome Atlas (TCGA). 15.2% patients (56 out of 368 samples) were identified as TP53 mutation. There was no difference in the frequency of TP53 mutation between TCGA and the clinical cohort (P = 0.17). No difference of OS was observed between TP53 mutation and TP53 wild-type (HR, 0.87; 95% CI, 0.55 to 1.37; P = 0.55, Supplemental Fig. S2a). Then we tested if TP53 mutation was associated with overall survival in patients with stage III or IV melanoma. There was no significant association between TP53 mutation and overall survival in stage III or IV (HR, 0.59; 95% CI, 0.31 to 1.12; P = 0.09, Supplemental Fig. S2b), even though there was a trend that TP53 mutation was associated with better overall survival. These results suggested that TP53 was a potential negative predictor for the clinical benefit of anti-CTLA-4 therapy in metastatic melanoma instead of a prognosis factor for melanoma.

### 3.5 Association Between TP53 Mutation and Immune-Related Gene Signature

**Fig. 2:**
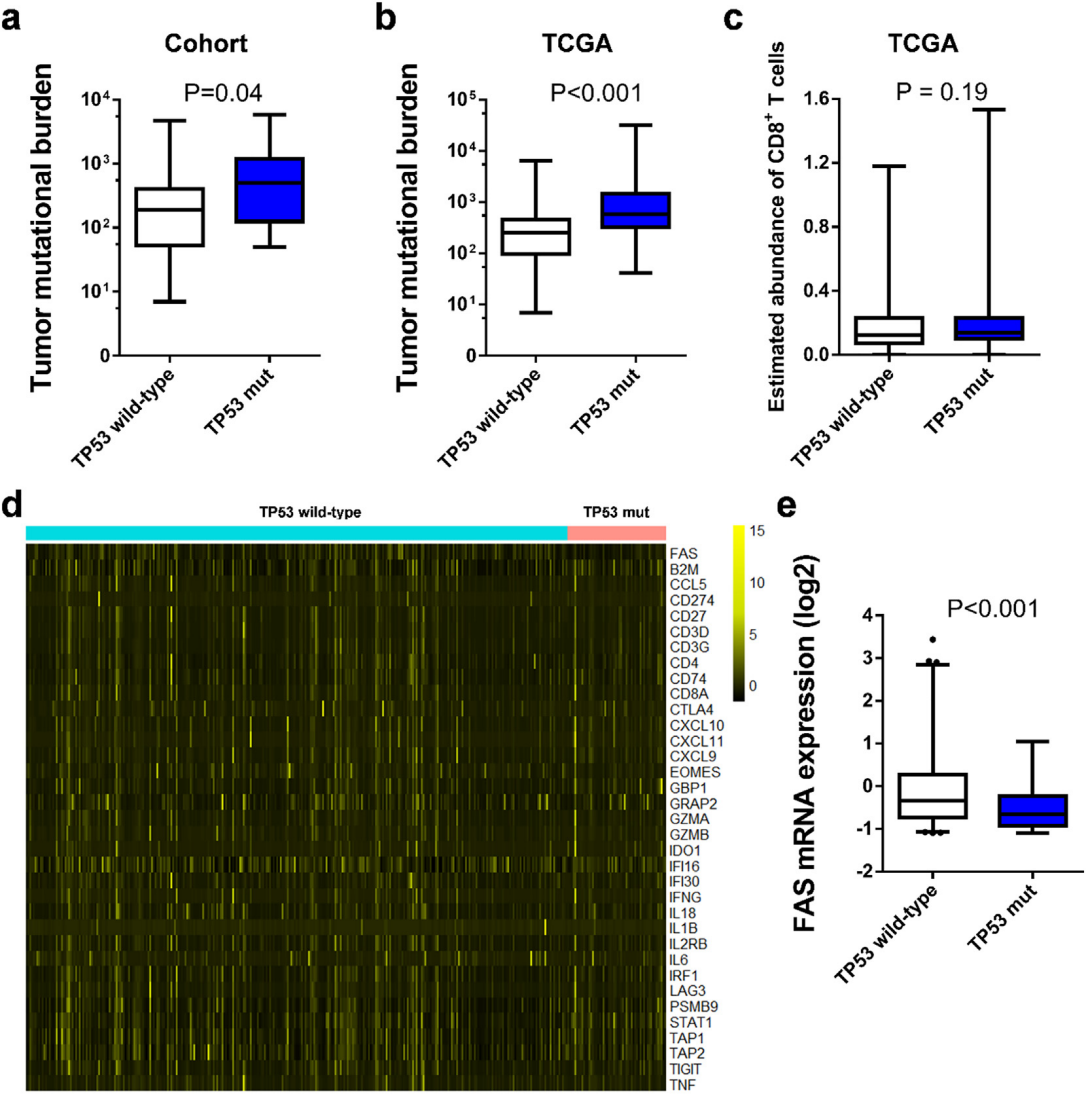
Possible mechanism of the association of TP53 mutation and clinical outcomes of anti-CTLA-4 therapy. (a) Comparison of tumor mutational load between patients with TP53 mutation and TP53 wild-type in the clinical cohort. (b) Comparison of tumor mutational load between patients with TP53 mutation and TP53 wild-type in TCGA. (c) Comparison of CD8^+^ T cell infiltration between patients with TP53 mutation and TP53 wild-type in melanoma (d) Heatmap depicting the mRNA expression of immune-related genes between patients with TP53 mutation and TP53 wild-type. (e) Comparison of mRNA expression of FAS between patients with TP53 mutation and TP53 wild-type in melanoma.

To further explore the underlying mechanism of TP53 mutation and poor survival of CTLA-4 blockade in melanoma, we associated TP53 mutation with TMB and immune-related gene signature. Patients with TP53 mutation had higher TMB compared to patients with wild-type TP53 (Fig. 2a, b), indicating that patients with TP53 mutation was supposed to acquire better response to anti-CTLA-4 therapy. While as shown above, TP53 mutation was an independent indicator of poorer outcomes beyond TMB status, suggesting the negative prognosis of TP53 mutation receiving anti-CTLA-4 treatment may attribute to other factors.

We further analyzed the association between CD8^+^ T cell infiltration and TP53 mutation, however no association was observed (P = 0.19, Fig. 2c). We then analyzed the mRNA data from TCGA, no difference of the expression of other immune related genes including B2M, CD274, CD8A, CTLA-4, CXCL10, CXCL11 and several chemokines was detected between two groups (Fig. 2d) except that the mRNA expression of FAS was lower in TP53 mutation than TP53 wild-type patients (FDR < 0.05, Fig. 2e) It has been demonstrated that TP53 may up-regulate FAS expression in tumor cells, which results in the induction of CTL-mediated apoptosis [11,12]. We supposed that the association between TP53 mutation and poor outcomes of anti-CTLA-4 in melanoma may be partially on account of the down-regulation of FAS mRNA expression by TP53 mutation, impeding CTL-mediated apoptosis of tumor cells.

## 4 Discussion

In the present study, we observed that TP53 mutation was associated with poorer PFS, OS and objective response or long-term benefit rate among patients with metastatic melanoma receiving anti-CTLA-4 therapy. The down-regulated mRNA expression of FAS was a possible underlying mechanism of association between TP53 mutation and poor outcome of anti-CTLA-4 therapy.

A previous study in lung adenocarcinoma treated with PD-1 blockade has demonstrated that TP53 mutation is associated with higher TMB, increased immune-related genes and prolonged PFS [15]. However, in this study, we observed the opposite predictive value of TP53 in melanoma. Indeed, TP53 mutation is also associated with higher TMB in melanoma, however, the multivariate regression model suggested the negative predictive value of TP53 mutation is independent of TMB status. Also, no association was observed between TP53 mutation and increased immune-related genes expression in melanoma. Instead, lower level of FAS mRNA was detected in TP53 mutated melanoma patients with false discovery rate <0.05, in harmony with a previous study [12]. Taken together, it is presumable that the down-regulation of FAS level by TP53 mutation impeded CTL-mediated apoptosis, limiting the benefit of anti-CTLA-4 therapy. However, the underlying mechanism needs to be further explored.

In addition to TP53 mutation, other biomarkers including TMB, LDH and stage were associated with clinical outcomes of anti-CTLA-4 therapy consistent with previous studies [7,16,19]. It was reported that abnormal LDH (>upper limit) predicts poorer prognosis in NSCLC [21], and in the present study, LDH-abnormal melanoma patients exhibited poorer PFS, OS and response in univariate and multivariate models. As for TMB, median TMB is associated with the objective response rate of ICBs among 27 tumor types [22]. Besides, TMB was also illustrated to be associated with clinical response to anti-CTLA-4 [16,23] and anti-PD-1 [7] in melanoma. In the present study, the association between TMB-high and clinical outcome was not consistent in several univariate analyses, possibly on account of the interference of other confounders containing LDH and stage. Also, the cut-off value of TMB may vary largely in different studies. In the present study, median was applied as the cut-off value and the statistical significance may be more pronounced with other cut-off values. Difficulties in determining the cut-off value of TMB limited its clinical application as a prognostic biomarker. The last but not the least we didn't observe the association between indel burden and clinical outcome in melanoma. Because the SNV neoantigen is much higher than frameshift neoantigen in melanoma [24], we supposed the SNV neoantigen still plays a vital role in melanoma even though in-del mutations are in particularly immunogenic.

This retrospective study has several limitations. First, this analysis was based on a public cohort of melanoma patients who underwent whole-exome sequencing, which might yield selection bias. Second, we defined any mutation in TP53 coding region as TP53 mutation, while the function of various genetic mutation of TP53 may vary remarkably and the underlying mechanism between TP53 and immunotherapy in melanoma should be further investigated. Third, PD-1 mAbs is indicated to be the standard therapy for the treatment of melanoma and whether TP53 mutation predicts response to anti-PD-1 should be further explored. However, the lack of increased immune-related genes expression in patients with TP53 mutation may suggest TP53 mutation is not a positive predictor of response to PD-1 blockade in melanoma. Forth, most patients of TCGA cohort were early stages, whose baseline characteristics may be different from the clinical cohort used in the present study. Finally, limited quantity of patients with TP53 mutation might restrict the application of the conclusions in the present study, which should be investigated with a larger sample size.

In conclusion, this study revealed that TP53 mutation may serve as a negative predictor of metastatic melanoma treated with anti-CTLA-4 therapy. This predictive value should be explored further in larger cohorts with prospective setting. Additional investigation is warranted to evaluate the underlying mechanisms between TP53 mutation and immunotherapy.

## Appendix A Supplementary Data

Supplementary material Image 1
