# Supplementary material for: TP53 Mutation as Potential Negative Predictor for Response of Anti-CTLA-4 Therapy in Metastatic Melanoma
Source: eBioMedicine. 2018 May 22;32:119–24. doi: 10.1016/j.ebiom.2018.05.019 (PMC6020711; doi:10.1016/j.ebiom.2018.05.019)
Supplement: Supplementary file 1 — Supplementary material [file mmc1.docx]

Table S1. Baseline characteristics and clinical outcomes of patients with TP53 mutation

| Patient | Age | Gender | Stage | TP53 mutation | BRAF  V600E | Overall survival  (months) | OS  event | Progression free survival  (months) | Progression  event | TMB | Response or long-term survival |
| --- | --- | --- | --- | --- | --- | --- | --- | --- | --- | --- | --- |
| Pat109 | 69 | Male | Stage 4 | Missense mutation | wild-type | 2.77 | Deceased | 0.63 | Progressed | 245 | nonresponse |
| Pat11 | 67 | Male | Stage 4 | Missense mutation | mutation | 26.33 | Censored | 4.20 | Progressed | 2058 | long-survival |
| Pat110 | 76 | Male | Stage 4 | Missense mutation | wild-type | 10.67 | Deceased | 2.43 | Progressed | 5976 | nonresponse |
| Pat162 | 55 | Female | Stage 4 | Missense mutation | wild-type | 7.03 | Deceased | 2.60 | Progressed | 51 | nonresponse |
| Pat37 | 47 | Female | Stage 4 | Missense mutation | wild-type | 2.33 | Deceased | 0.77 | Progressed | 127 | nonresponse |
| Pat45 | 68 | Male | Stage 4 | Missense mutation | wild-type | 2.97 | Deceased | 2.03 | Progressed | 931 | nonresponse |
| Pat148 | 35 | Female | Stage 4 | Nonsense mutation | mutation | 2.80 | Deceased | 1.60 | Progressed | 129 | nonresponse |
| Pat71 | 63 | Male | Stage 4 | Nonsense mutation | wild-type | 4.57 | Deceased | 3.27 | Progressed | 660 | nonresponse |
| Pat74 | 73 | Male | Stage 4 | Nonsense mutation | wild-type | 6.43 | Deceased | 2.67 | Progressed | 518 | nonresponse |
| Pat100 | 70 | Male | Stage 4 | Splice site | wild-type | 12.00 | Deceased | 3.70 | Progressed | 522 | nonresponse |


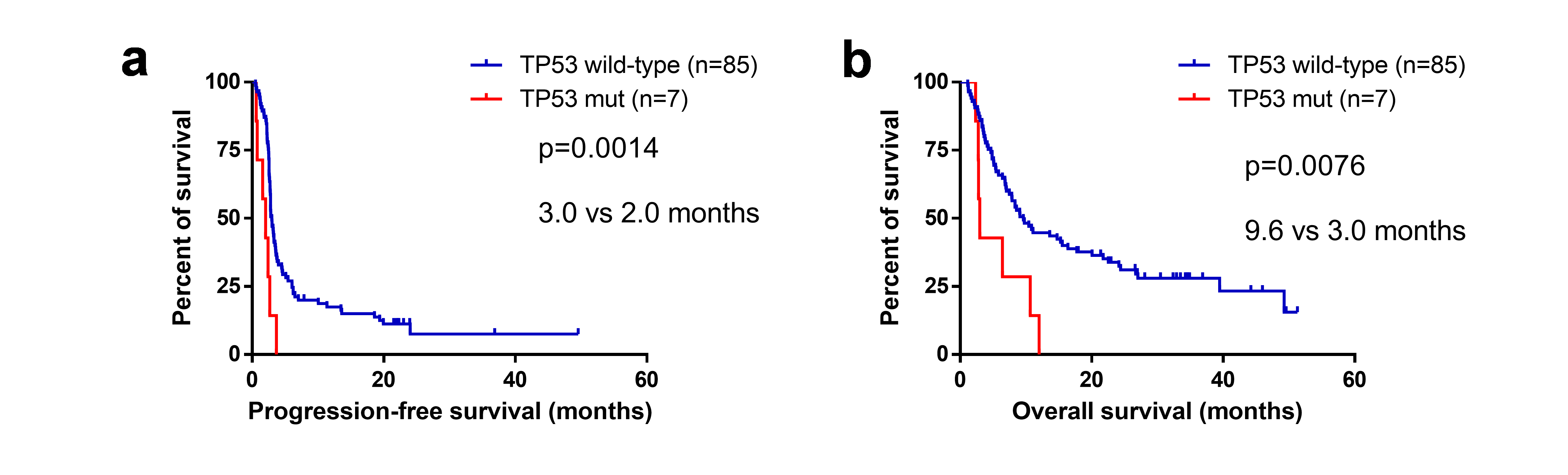


**Figure S1. Association between TP53 mutation status and survival outcomes in skin melanoma subgroup.** (a) Kaplan-Meier survival curves of progression-free survival comparing patients with TP53 wild-type and patients with TP53 mutation in skin melanoma. (b) Kaplan-Meier survival curves of overall survival comparing patients with TP53 wild-type and patients with TP53 mutation in skin melanoma.


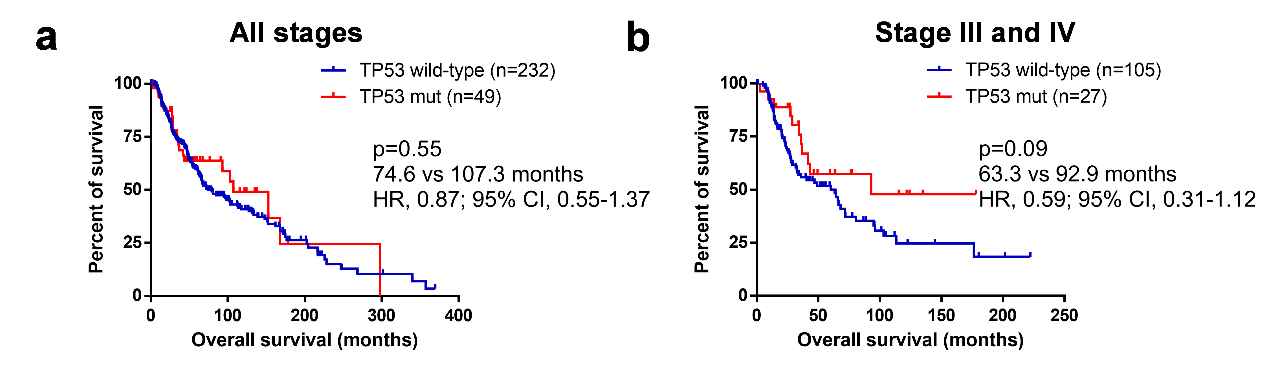


**Figure S2. Association between TP53 mutation status and overall survival in patients with skin melanoma from TCGA.** (a) Kaplan-Meier survival curves of progression-free survival comparing patients with TP53 wild-type and patients with TP53 mutation in patients with skin melanoma from TCGA. (b) Kaplan-Meier survival curves of progression-free survival comparing patients with TP53 wild-type and patients with TP53 mutation in patients with skin melanoma of stage III or IV from TCGA.
